# The associations between emotional neglect and positive mental health: the mediating role of stress-is-enhancing mindset and the moderating role of stress-is-debilitating mindset

**DOI:** 10.3389/fpsyt.2026.1834490

**Published:** 2026-07-24

**Authors:** Hongchao Wang, Mengxuan Li, Yu Gao, Min Zou, Haizhou Leng, Chao Gao

**Affiliations:** 1 School of Psychology, Shandong Second Medical University, Weifang, China; 2 School of Educational Science, Yili Normal University, Ghulja, China; 3 School of Education, Weifang University, Weifang, China

**Keywords:** cross-sectional study, emotional neglect, positive mental health, stress-is-debilitating mindset, stress-is-enhancing mindset

## Abstract

**Objective:**

This study aimed to investigate the associations among emotional neglect (EN), stress-is-enhancing mindset (SIEM), stress-is-debilitating mindset (SIDM), and positive mental health (PMH).

**Methods:**

The cross-sectional survey was employed in this study. A sample of 2150 college students (826 males) completed the emotional neglect subscale of the Childhood Trauma Questionnaire Short Form, the Stress Mindset Measure and the Positive Mental Health Scale. The PROCESS macro version 4.2 for SPSS was used to examine the moderated mediation model.

**Results:**

EN negatively predicted PMH (*p* < 0.001). SIEM mediated the association between EN and PMH. Moreover, SIDM (*β* = 0.04, *p* < 0.001) not merely moderated the relationship between EN and PMH, but also moderated the link between EN and SIEM (*β* = 0.04, *p* < 0.001).

**Conclusion:**

EN has a direct negative link to PMH and an indirect negative link to PMH through SIEM. Importantly, high SIDM mitigates the direct negative relation between EN and PMH. Moreover, the indirect negative effects of EN on PMH via SIEM diminish as SIDM increases, and the mediation pathway of SIEM is neutralized under high SIDM. These findings preliminary reveal the interplay between SIEM and SIDM in post-traumatic cognitive processing, offering new insights into the non-linear processes of psychological adaptation following early adversity.

## 1 Introduction

Mental health is the cornerstone of health and quality of life, and it underpins daily functioning and serves as a vital resource for the healthy operation of individuals, families, communities, and society at large (1). The dual-factor model states that mental health comprises two distinct dimensions of mental ill-being (e.g., anxiety) and mental well-being (e.g., life satisfaction) (2, 3), and emphasizes that it is necessary to integrate the positive and negative indicators to comprehensively assess mental health (2). Unlike mental ill-being, positive mental health (PMH), also known as mental well-being, mainly involves emotional, psychological, and social well-being (4). PMH can enhance psychological adaptation (5) and alleviate depression (6). As a group situated in emerging adulthood, college students are at a critical stage for identity formation and value development (7), their mental health are relatively vulnerable. Therefore, it holds a significant practical importance to explore the influencing factors of college students’ PMH for their healthy development.

The multilevel model of mental health argues that, both in childhood and adulthood, supportive social environments across the lifespan affect mental health (3). The stable and emotionally fulfilling social relationships are the crucial prerequisite for mental health promotion (3), implying that for individuals growing up in a social environment lacking emotional feedback, their mental health may be at risk. Emotional neglect (EN), defined as the failure to respond to children’s emotional needs (8), is precisely a missing form of supportive social circumstance. In such an environment, the higher the EN perceived by an individual, the more severe their internalized symptoms become (9). Hence, EN is a critical risk factor in the development of mental health. As the most frequently reported type of childhood maltreatment (10), EN is negatively associated with well-being (10–13). Research has shown that emotional regulation difficulties (10), negative emotions (e.g., depression and anxiety) (12), less positive coping styles (11), or lower self-esteem (12) plays a mediating role in the negative associations between EN and PMH. However, the existing mechanisms mainly focus on the reasons why EN has negative impacts on PMH, and they have difficulties in clarifying the mechanism when individuals with EN exhibit higher levels of PMH. Consequently, the present understanding regarding the relationship between EN and PMH is limited.

Post-traumatic growth (PTG) refers to the positive psychological changes that emerge during a process wherein individuals actively struggle with trauma (14, 15). PTG is related to positive affection (15). Positive affection belongs to the category of emotional well-being, thereby PTG may be partially linked to PMH. According to the PTG model, positive cognitive reconstructing is the core mechanism of PTG—individuals reinterpret traumatic experiences and integrate them into life narratives with growth significance (15). Hence, a reasonable speculation is that individuals with EN may reinterpret the meaning of EN, achieve personal growth, and thereby foster PMH.

However, EN is reported to negatively predict PTG (16, 17), suggesting that EN may not influence PMH via PTG. It is possible that the PTG model is primarily designed to explain growth following a discrete, major traumatic event, whereas the nature of EN is a chronic stressor that persists throughout development and may therefore exert its influence via a distinct underlying mechanism. As Murphy and Harris (18) argue that, the traditional PTG model cannot explain the adaptation process of chronic stress. With semi-structured interviews, they found that the positive adaptation of patients with chronic fluctuating diseases manifests as a dynamic cycle between psychological distress and self-flexibility, rather than a linear progression (18). Although the study reveals the unique process of positive adaptation under chronic stress, it fails to uncover the underlying mechanisms driving individuals to persistently engage in self-flexibility.

In fact, repeated exposure to a specific type of event gradually shapes a relatively stable cognitive schema (19). Extending this logic to the study by Murphy and Harris (18) suggests that, when confronted with chronic stressors, the repeated and successful utilization of self-flexibility to manage trauma-induced psychological distress may, through this iterative process, gradually foster a relatively stable positive meta-cognitive belief regarding the nature of stressors—specifically, that active coping can lead to growth. This positive meta-cognitive belief falls under the category of higher-order meta-cognitive monitoring, and may act as an internal motivational force to drive individuals to continually mobilize self-flexibility when facing the ongoing distress related to trauma. However, prior studies focused on the mediating role of positive cognitive restructuring between chronic stressors and PTG (20–22), whereas the potential motivational mechanism of positive meta-cognitive belief about the nature of trauma remains unexplored within the PTG model.

Recently, the development of stress mindset theory provides a new view for understanding this mechanism. Stress mindset refers to a meta-cognitive belief about the nature of stress, reflecting the extent to which individuals believe that stress can promote growth or lead to impairment (23, 24). This construct consists of two orientations: a stress-is-enhancing mindset (SIEM), which holds that stress can promote growth, and a stress-is-debilitating mindset (SIDM), which holds that stress impairs physical and mental functioning. SIEM and SIDM are two related but relatively independent constructs and trigger distinct psychological and physiological effects (23, 24). This two-dimensional structure, which treats SIEM and SIDM as two distinct dimensions rather than opposite ends of a single continuum, has been widely verified in previous research (e.g., 24–27).

Evidence has shown that SIEM was positively correlated with PTG (28) and directly enhanced positive affect (24). Importantly, SIEM is indirectly and positively linked to well-being via adaptive coping strategies (e.g., positive reframing) (25, 29). This may position SIEM as a key mechanism via which individuals with SIEM employ adaptive coping strategies to enhance well-being. For individuals with EN, the fewer positive coping styles are positively related to their lower PMH (11). Thus, when confronted with EN, those with SIEM are likely to employ adaptive coping strategies to show a positive link to PMH. That is, SIEM may play a mediating role in the relationship between EN and PMH.

However, SIDM can directly enhance negative affect (24) and indirectly increase loneliness via maladaptive coping strategies (30). Importantly, Long found that the negative effect of adverse childhood experiences (ACEs) on resilience (an indicator of PMH) was enlarged under high SIDM, whereas the moderating effect of SIEM was non-significant (31). This suggests that SIDM may function as an active inhibitory factor that directly amplifies the virulence of ACEs. In contrast, the potential of SIEM for fostering resilience is more likely to manifest only when individuals are not dominated by SIDM. That is, the effect of SIEM can be “suppressed” by SIDM, making its protective effect unobservable in individuals with high ACEs.

Indeed, a longitudinal study revealed that SIDM negatively predicted subsequent SIEM (27). This suggests that individuals can simultaneously hold two different stress mindsets, and that SIDM is an important antecedent influencing changes in SIEM. Cognitive dissonance theory further posits that, when individuals simultaneously hold two or more contradictory beliefs (e.g., SIEM vs. SIDM), they experience psychological maladaptation (32). To restore cognitive consistency, individuals tend to modify the cognitive content that conflicts with their dominant belief by adjusting, avoiding, or diminishing the conflicting cognition (32). Therefore, when SIDM predominates, the strength of SIEM may be attenuated, rendering it unable to function effectively. This may explain why SIDM but not SIEM moderates the relationship between ACEs and resilience.

As a type of ACEs, EN shows a negative link to PMH via reduced positive coping styles (11) and increased depression and anxiety (12). According to the finding of Long (31) and the cognitive dissonance theory (32), when SIDM predominates under exposure to EN, the levels of SIEM may be lower. Consequently, we hypothesize that the negative links of EN to PMH may be amplified through two pathways. First, SIDM may directly intensify the emotional distress linked to EN and erode PMH. Second, SIDM may block the utilization of adaptive coping strategies connected with SIEM to enhance the detrimental link of EN to PMH. Specifically, SIDM moderates the first stage of the mediating pathway of SIEM between EN and PMH (EN→SIEM). When SIDM is high, EN shows a negative link to PMH through low SIEM; when SIDM is low, the negative link of EN to SIEM is weakened, thereby mitigating the impairment of PMH.

Notably, the current study employed an absolutely new and independent sample, and there was no participant overlap with our another study which aimed to investigate whether both SIEM and SIDM moderated the mediating pathway of positive mental health literacy (PMHL) in the link between childhood trauma and PMH within the transactional model of stress and coping (33). The present study examined whether the mediating pathway of SIEM in the association between EN and PMH exhibited heterogeneity depending on individuals’ SIDM within the PTG model.

**Figure 1:**
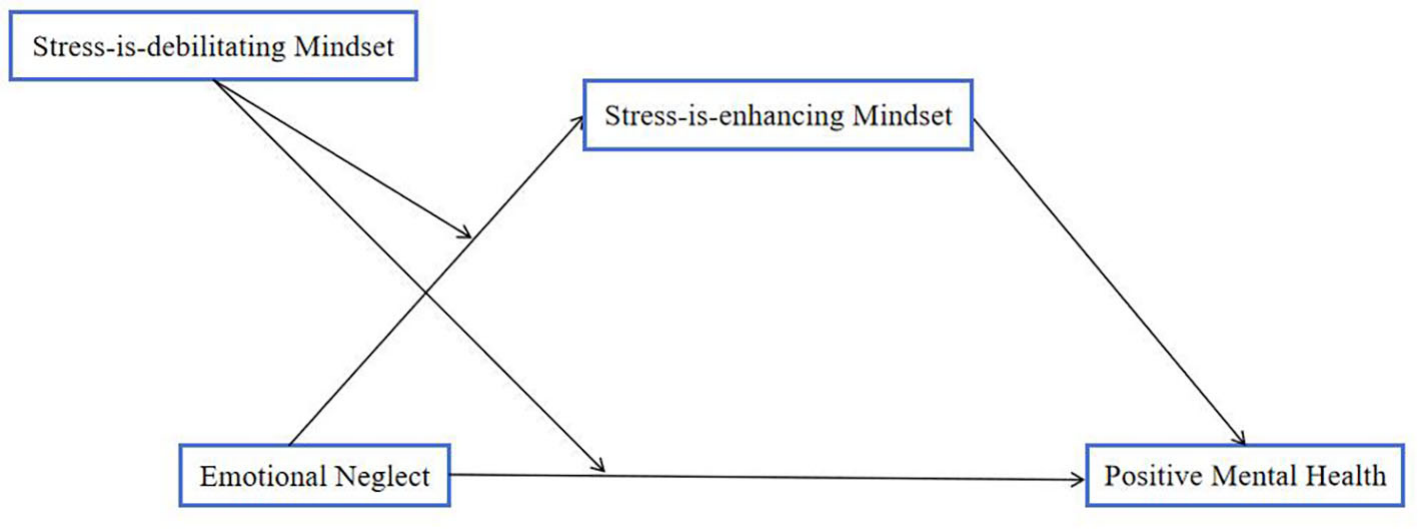
The theory framework.

In summary, this study integrates the PTG model with stress mindset theory to explore whether SIEM buffers the negative effects of EN on PMH, and to examine the moderating role of SIDM in this mediating mechanism. By preliminary elucidating the interactive patterns between SIEM and SIDM, this study preliminary intends to provide empirical evidence for the synergistic influence of these two mindsets on adaptation to chronic stress and enrich the theoretical landscape of the stress adaptation field. Furthermore, we introduce meta-cognitive dimension into PTG field to extend the applicability of the PTG model to chronic relational trauma and address the theoretical limitation of the predominant focus on acute traumatic events. At the practical level, this study aims to identify the meta-cognitive targets for precise intervention in EN, offering a theoretical foundation for clinical practice to facilitate individuals’ transformation of stress into growth. The theoretical framework is depicted in **Figure 1** and the hypotheses are as follows.

Hypothesis 1: EN is negatively associated with PMH;
Hypothesis 2: SIEM mediates the relationship between EN and PMH;
Hypothesis 3: SIDM amplifies the negative association between EN and PMH;
Hypothesis 4: SIDM enhances the negative association between EN and PMH via SIEM.

## 2 Methods

### 2.1 Study sample

This study was approved by the Ethics Committee of Shandong Second Medical University. In accordance with the Helsinki Declaration, the current convenience-sampling study collected data between September 2023 and April 2024 from four universities in Northeast and East China. Four teachers from participating universities assisted with recruitment by introducing the study’s objectives and the instructions for completing the questionnaire during class breaks, emphasizing voluntary participation and its independence from academic evaluation. After obtaining informed consent, a questionnaire QR code generated via the Wenjuanxing platform was distributed to college students through social networks such as WeChat and QQ groups.

Prior to participation, individuals were informed of the study’s purpose and provided the informed consent. To facilitate data quality assurance, each respondent was asked to create a self-defined unique temporary username consisting of Chinese characters, letters or numbers, containing no any personal identification information (e.g., names or student IDs), thereby ensuring participant anonymity. These usernames were used solely to identify and eliminate duplicate entries and were removed upon completion of data cleaning. A total of 3, 000 responses were acquired. After removing duplicate submissions by cross-verifying demographic data with usernames, retaining only the initial submission, 2, 369 unique questionnaires were obtained.

The eligibility criteria for inclusion were as follows: (1) complete demographic information (e.g., age, hometown); (2) aged between 18 and 25 years; (3) correct responses to the attention-check item (e.g., please select “A” in this question); and (4) the 5th percentile of response times in the valid sample (N = 2278, after excluding invalid cases for completeness, age range, and attention-check correctness) was 1.93 minutes. Thus, the minimum completion time of 2 minutes (slightly above the 5th percentile) was set to conservatively exclude responses likely reflecting inattentive or overly rapid answering. According to these criteria, 219 invalid responses were removed from the 2, 369 unique responses, and 2, 150 valid questionnaires were retained, yielding an effective response rate of 90.76%.

Notably, the sample had no fourth-year or higher-year students, and sophomores represented the majority. This imbalance stemmed from the convenience sampling method, which relied on instructors teaching lower-division courses and social media dissemination primarily within the second-grade networks. The age range of the current sample was between 18 and 25 years, with a mean age of 19.82 years, their major involved the medical, engineering, and education. The distribution characteristics of the sample in this study see **Table 1**.

**Table 1:** The distribution characteristics of the valid samples (n = 2150).

Variable		Frequency	Percent
Gender	Male	826	38.42
	Female	1324	61.58
Age	18∼21	2024	94.14
	22∼25	126	5.86
Hometown	City	910	42.33
	Countryside	1240	57.67
Grade	Grade 1	309	14.37
	Grade 2	1796	83.53
	Grade 3	45	2.10

### 2.2 Measures

#### 2.2.1 Emotional neglect scale

EN was measured using the emotional neglect subscale of the Childhood Trauma Questionnaire-Short Form (CTQ-SF) (34). The Chinese version of the CTQ-SF was revised by Zhao et al. (35), and higher reliability and validity were reported in Chinese undergraduates (36). EN subscale contains 5 items (e.g., felt loved), with a 5-point Likert scale ratings ranging from 1 (never) to 5 (always). The total score of the subscale ranges from 5 to 25, higher the total score indicates more EN experiences. In this study, the Cronbach’s alpha of EN subscale was 0.804.

#### 2.2.2 Stress mindset measure (SMM)

Stress mindset was measured using the SMM (23). The Chinese version of the SMM revised by He et al. (37) has good reliability and validity among Chinese university students. The SMM consists of two subscales: stress-is-enhancing mindset (SIEM; e.g., experiencing stress improves my health and vitality) and stress-is-debilitating mindset (SIDM; e.g., experiencing stress depletes my health and vitality). Each subscale comprises 4 items rated on a 5-point Likert scale ranging from 0 (strongly disagree) to 4 (strongly agree). The total score for each subscale ranges from 0 to 16, with higher scores indicating a stronger level of the corresponding stress mindset. In this study, the Cronbach’s alpha for SIEM and SIDM subscales were 0.859 and 0.808, respectively.

#### 2.2.3 Positive mental health scale (PMHS)

PMH was measured using the PMHS (38). The Chinese version of PMHS revised by Ding et al. (39) shows good reliability and validity among Chinese adults aged from 19 to 66 years. The PMHS consists of 9 items (e.g., I enjoy my life), with a 4-point Likert scale ratings ranging from 1 (not true) to 4 (true), the total score ranges from 9 to 36, and the higher the total score, the higher the levels of PMH. The Cronbach’s alpha of PMHS in current sample was 0.951, comparable to the value of 0.95 reported in the original sample. The confirmatory factor analysis (CFA) showed that the single-factor structure of PMHS exhibited good fit (χ²/df = 14.880, *p* < 0.001, RMSEA = 0.080, CFI = 0.949, TLI = 0.931, SRMR = 0.026), preliminarily supporting the applicability of PMHS in the current sample.

### 2.3 Data analysis

Data were analyzed using SPSS 26.0. Kolmogorov-Smirnov test indicated that all continuous variables deviated significantly from normal distribution (*ps* < 0.001). Consequently, the bivariate associations were examined using Spearman’s rank correlation analysis. Differences between groups based on gender and hometown were assessed using the Mann-Whitney U test, while differences across grades were analyzed using the Kruskal-Wallis H test.

A systematic review proposed that, gender, developmental stage (age/grade), and neighborhood environments (hometown) moderated the effects of early life stress (including emotional neglect) on child’s mental health (40). Empirical evidence showed that neighborhood safety was only associated with internalizing symptoms (IS) and externalizing symptoms (ES) of male adolescents, and the connection between family conflicts and ES was strengthened among male adolescents (41), indicating that gender plays a key moderating role between early-life stress and mental health. Thus, to obtain unbiased estimates of the central mediation and moderated mediation paths, these demographic variables above were included as covariates.

The cross-sectional design was used in this study to preliminarily test the validity of the proposed model. Therefore, the PROCESS macro version 4.2 for SPSS (42) was employed to examine the mediating role of SIEM in the relationship between EN and PMH and whether SIDM moderated the mediating effect of SIEM and the direct pathway from EN to PMH. To test the significance of these effects, the bias-corrected percentile bootstrapping method with 5, 000 resamples was used to estimate 95% confidence intervals. The direct and indirect effects were considered statistically significant if the 95% confidence intervals did not include zero.

To reduce multicollinearity, the “only continuous variables that define products” option in the PROCESS was selected, which automatically mean-centers continuous variables involved in interaction terms. HC1 (Hinkley) standard errors were used for heteroscedasticity-consistent inference, and the conditional effects were examined at the mean and at plus and minus one standard deviation from the mean. The Harman single-factor test was employed to assess common method bias (CMB), and the *Mplus* 8.3 was used to conduct the CFA.

## 3 Results

### 3.1 Common method bias

The Harman single-factor test showed that four factors with eigenvalues greater than 1 were extracted. The first factor accounted for 34.20% of the total variance, which was less than the commonly recommended threshold of 40%. This suggests that CMB is not significant in this study.

### 3.2 Descriptive statistic and correlation analysis

Mann-Whitney U Test showed that the gender differences among SIEM, PMH and EN were significant (*ps* < 0.01), the hometown differences among PMH and EN were significant (*ps* < 0.001). Kruskal-Wallis H Test showed that the grade differences among SIEM, SIDM, PMH and EN were significant (*ps* < 0.01). Spearman’s correlations analysis showed that age was significantly linked to PMH (*p* < 0.05). These results align with the theoretical expectations (40, 41). The detailed results were presented in **Table 2**.

**Table 2:** Means, standard deviations and correlations analysis results of the key variables.

Variables	1	2	3	4	5
1. Emotional neglect	1				
2. Stress-is-enhancing mindset	−0.12***	1			
3. Stress-is-debilitating mindset	0.11***	−0.29***	1		
4. Positive mental health	−0.40***	0.26***	−0.12***	1	
5. Age	0.01	0.01	−0.01	0.05*	1
Mean	8.62	9.95	6.89	26.86	19.82
Standard deviation	3.70	2.79	3.02	6.47	1.01

n = 2150, *p < 0.05, ***p < 0.001.

### 3.3 The mediation effect test

The mediating role of SIEM in the relation between EN and PMH was examined using the Model 4 of the PROCESS macro version 4.2 (age, gender, hometown and grade as covariates). Results showed that EN negatively predicted SIEM (*β* =−0.12, *t* = −4.59, *p* < 0.001) and PMH (*β* =−0.34, *t* = −14.53, *p* < 0.001), SIEM positively predicted PMH (*β* = 0.22, *t* = 9.10, *p* < 0.001), indicting that SIEM may serve as a mediator in the link between EN and PMH. The results see **Table 3**.

**Table 3:** The results of the mediation model.

Independent variables	Dependent variable: stress-is-enhancing mindset		Dependent variable: positive mental health	
	*β*	*t*	*β*	*t*
Model summary	R^2^ = 0.04	F = 11.56***	R^2^ = 0.21	F = 70.50***
Emotional neglect (EN)	−0.12	−4.59***	−0.34	−14.53***
Stress-is-enhancing mindset (SIEM)	–	–	0.22	9.10***
Age	0.001	0.05	0.06	2.72**
Gender	0.15	6.58***	0.08	3.85***
hometown	0.03	1.48	−0.04	−2.22*
Grade 1	−0.06	−2.58*	−0.06	−2.69**
Grade 3	−0.05	−1.82	−0.05	−2.24*
Indirect effect of X (EN) on Y (PMH) via mediator (SIEM)				
	Effect	BootSE	BootLLCI	BootULCI
Stress-is-enhancing mindset (SIEM)	−0.04	0.01	−0.07	−0.03

n = 2150, *p < 0.05, **p < 0.01, ***p < 0.001, grade 2 was the reference. Standardized regression coefficients were reported.

### 3.4 The moderated mediation effect test

The moderating effect of SIDM was examined using Model 8 in the PROCESS macro version 4.2 (age, gender, hometown and grade as covariates). The results indicated that the interaction effects between SIDM and EN on SIEM (*β* = 0.04, *t* = 4.97, *p* < 0.001) and PMH (*β* = 0.04, *t* = 3.80, *p* < 0.001) were significant. The index of moderated mediation was 0.02 (BootSE = 0.004, BootLLCI = 0.009, BootULCI = 0.024). The detailed results were summarized in **Table 4**.

**Table 4:** The results of the moderated mediation model.

Variables	Dependent variable: stress-is-enhancing mindset			Dependent variable: positive mental health		
	*β*	*SE*	*t*	*β*	*SE*	*t*
Emotional neglect (EN)	−0.09	0.02	−5.25***	−0.62	0.04	−16.35***
Stress-is-enhancing mindset (SIEM)	-	-	-	0.46	0.06	8.41***
Stress-is-debilitating mindset (SIDM)	−0.15	0.03	−4.67***	−0.002	0.05	−0.05
EN × SIDM	0.04	0.01	4.97***	0.04	0.01	3.80***
Age	0.01	0.06	0.12	0.37	0.13	2.80**
Gender	0.86	0.13	6.74***	1.06	0.27	3.87***
Hometown	0.15	0.12	1.26	−0.53	0.26	−2.07*
Grade 1	−0.40	0.19	−2.08*	−1.15	0.38	−3.02**
Grade 3	−0.69	0.46	−1.49	−2.24	1.11	−2.02*
Model summary	R^2^ = 0.09		F = 17.37***	R^2^ = 0.21		F = 59.51***

n = 2150, *p < 0.05, **p < 0.01, ***p < 0.001, grade 2 was the reference, unstandardized regression coefficients were reported.

**Figure 2:**
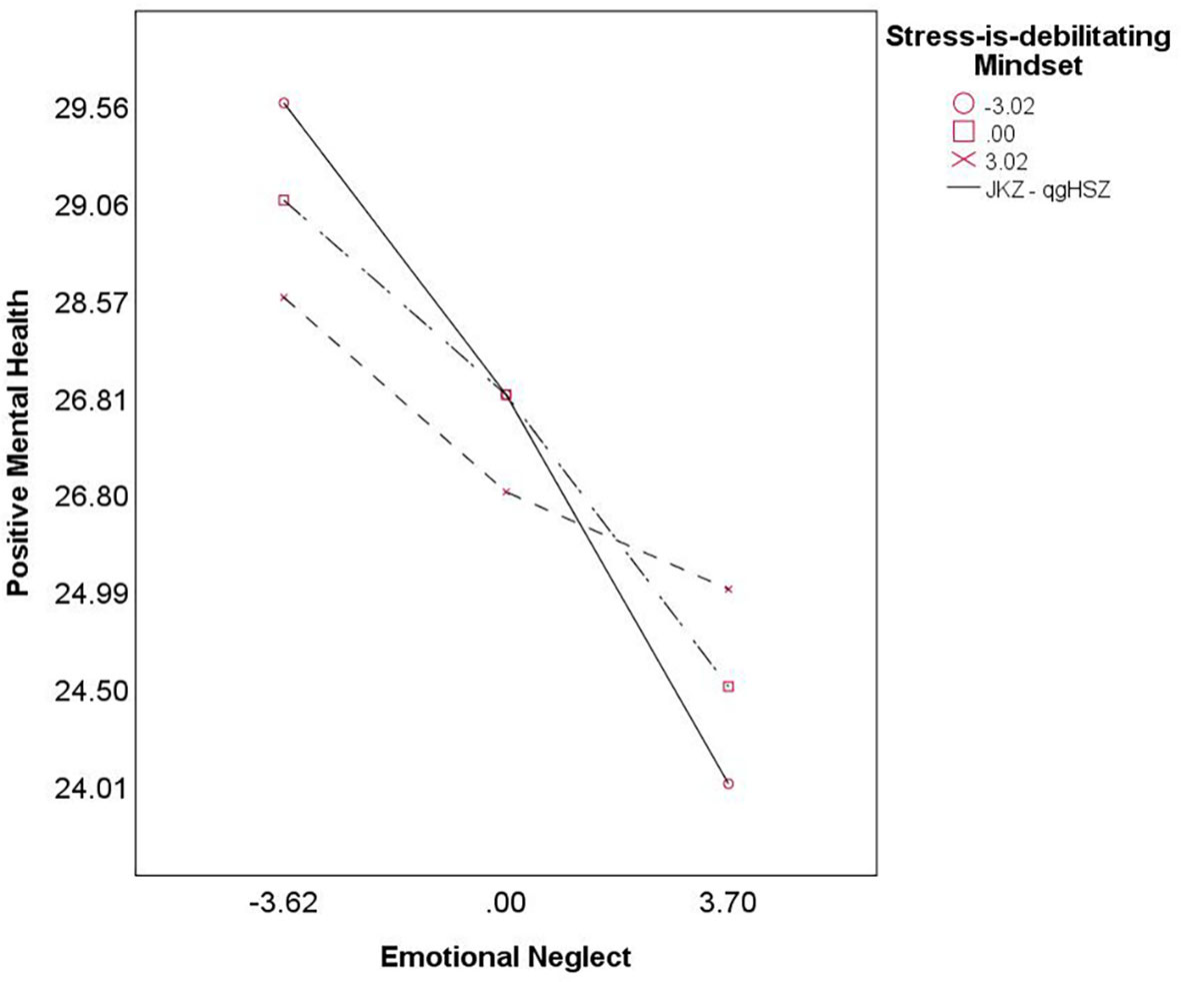
Stress-is-debilitating mindset (SIDM) as a moderator in the relationship between emotional neglect (EN) and positive mental health (PMH).

**Figure 3:**
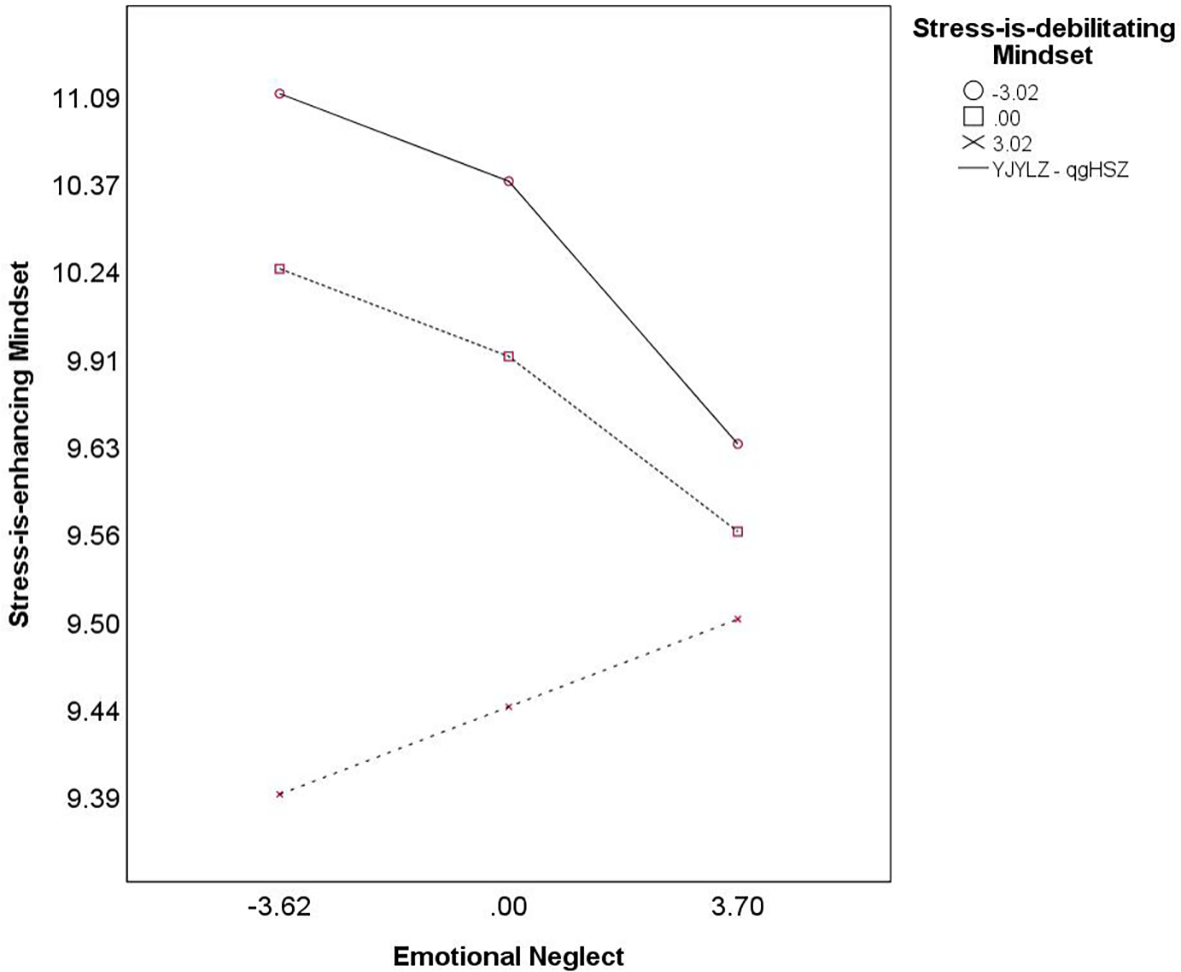
Stress-is-debilitating mindset (SIDM) as a moderator in the relationship between emotional neglect (EN) and stress-is-enhancing mindset (SIEM).

The simple slope analysis revealed that the negative links of EN to both PMH and SIEM were moderated by SIDM. As illustrated in **Figure 2**, the negative association between EN and PMH was weakened as SIDM increased. **Figure 3** showed that the negative relation between EN and SIEM was attenuated from lower levels of SIDM (SIDM scores were 1 standard deviation below the mean) to the mean level. However, when SIDM was at a higher level (SIDM scores were 1 standard deviation above the mean), the negative relation between EN on SIEM became non-significant. These findings indicate that SIDM attenuates the negative associations between EN and both SIEM and PMH.

**Figure 4:**
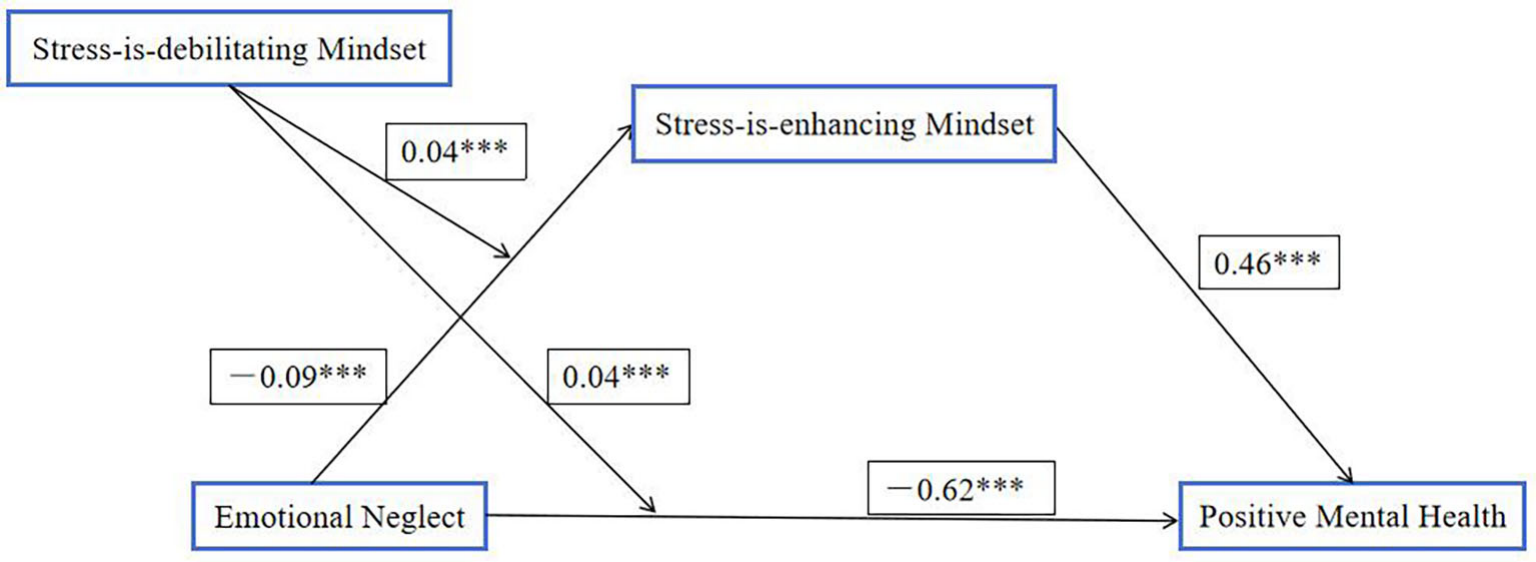
Results of the theoretical model (unstandardized regression coefficients were reported).

Importantly, the indirect effect of EN on PMH via SIEM was moderated by SIDM. Consistent with the patterns observed in the first stage of the mediation model, the indirect effect varied across levels of SIDM. At low levels of SIDM (effect =−0.09, BootSE = 0.02, BootLLCI =−0.13, BootULCI =−0.06) and mean levels of SIDM (effect =−0.04, BootSE = 0.01, BootLLCI =−0.06, BootULCI =−0.02), the indirect negative effects were significant. However, at high levels of SIDM, the indirect effect was not significant (effect = 0.01, BootSE = 0.01, BootLLCI =−0.02, BootULCI = 0.03). The overall theoretical model is depicted in **Figure 4**.

## 4 Discussion

To extend the applicability of the PTG model in the field of chronic stress, this study integrates the PTG model with stress mindset theory to construct a moderated mediation model centered on meta-cognitive beliefs. The results indicated that SIEM mediates the negative link between EN and PMH. Moreover, SIDM moderates the negative association between EN and PMH and the negative link between EN and SIEM. The study reveals that EN not only directly and negatively predicts PMH, but also indirectly and negatively predicts PMH via SIEM, while SIDM may modulate the direct and indirect negative link intensity between EN and PMH.

### 4.1 The relationship between emotional neglect and positive mental health

Hypothesis 1 was supported by the finding that EN negatively predicted PMH, indicating that EN was detrimental to the maintenance of PMH. This finding is consistent with previous research (10–13), suggesting that EN has a negative effect on PMH. The multilevel model of mental health believes that, an emotionally sufficient social environment is crucial for mental health (3). Accordingly, EN functions as a chronically emotionally insufficient social environment, which constitutes a chronic stressor and may be a risk predictor of PMH. This interpretation aligns with evidence that even daily stressor (e.g., school-related worries) is negatively linked to positive affect (43). However, unlike daily stressors with visible immediate impacts, the immediate impacts of EN are less visible (8). Thus, EN deserves special attention as a more concealed and persistent risk factor for PMH.

However, the present finding is contrary to the finding that EN facilitates PTG (14). Actually, PMH and PTG are two distinct constructs. PMH is a functional state involving social and psychological well-being (1), which may require the maintenance of a positive emotional environment. In contrast, PTG is an adaptive outcome following trauma and is not equivalent to an enhancement of well-being, but may instead represent a process of seeking meaning in adversity (44). Recent evidence has confirmed that EN negatively predicts PTG (16, 17). Combined with the current finding, this indicates that although EN has a detrimental relationship with both PTG and PMH, PTG and PMH may have essential differences in the mechanisms and connotations in the negative relation to EN.

### 4.2 The mediating role of stress-is-enhancing mindset

We found that EN negatively predicted SIEM, while SIEM positively predicted PMH. First, the negative association between EN and SIEM parallels the finding that EN negatively predicts positive time perspective (re-interpretation of time perception) (17). Together, these results suggest that EN is negatively associated with both positive meta-cognitive belief and positive cognitive strategies. Second, SIEM attenuates the negative link between EN and PMH, this is consistent with previous research demonstrating SIEM enhances positive emotional outcomes (24). Thus, our finding underscores the potential protective role of SIEM in undergraduates’ PMH.

Furthermore, our results revealed that SIEM mediated the relation between EN and PMH, this finding supports Hypothesis 2 and the PTG model (15). Specifically, the PTG model posits that, the intentional rumination on trauma can induce the positive cognitive restructuring, ultimately leading to PTG (15). EN, as a form of absent emotional reciprocity, is typically connected with lower levels of self-awareness and emotional confidence (10). Thus, individuals with EN may constantly experience emotional distress, which may induce an increase in repeated and voluntary reflection on EN. As a result, the SIEM regarding the nature of EN may be gradually developed. In return, SIEM is positively associated with challenge appraisals and adaptive coping strategies (25, 29, 45). Importantly, negative and positive self-conscious emotions (e.g., shame, guilt, pride) form a network that mutually influences behavioral outcomes (46). Therefore, when facing the emotional distress associated with EN, SIEM may buffer negative emotions and increase positive emotions by modulating this self-conscious emotion network, ultimately contributing to well-being.

Our findings suggested that college students with SIEM may exhibit high levels of PMH even if they had the experiences of EN, indicating that SIEM can be the critical protective factor in managing the adverse effects of EN on PMH. To some extent, the present finding extends the explanatory boundaries of the PTG model from a meta-cognitive perspective. Moreover, this study also provides a potential explanation for the proposed mechanism that self-flexibility facilitates PTG among patients with chronic fluctuating illnesses (18). However, PTG does not imply enhanced well-being or reduced distress, the significant growth can only be attained by substantial subjective distress (44). Therefore, whether SIEM promotes the transition from PTG to PMH, and whether PTG depends more on meta-cognitive appraisal rather than on distress itself require further validation.

### 4.3 The moderating role of stress-is-debilitating mindset

#### 4.3.1 The moderating role of stress-is-debilitating mindset between emotional neglect and positive mental health

Hypothesis 3 was rejected by the finding that SIDM positively moderated the association between EN and PMH, suggesting that SIDM may diminish the negative link between EN and PMH. In other words, when individuals have high SIDM, EN counterintuitively predicts the stronger PMH.

In our study, the protective effect of SIDM on the PMH in individuals with EN may not be an anomaly. This may reveal that SIDM’s protective function paradoxically depend on stressor chronicity. A recent longitudinal study revealed a similar phenomenon. Zhang et al. (47) found that, social mobility belief (a positive mindset) counterintuitively aggravates depression in adolescents who have high allostatic load (the cumulative physiological cost of early adversities) and are confronted with bullying. They proposed that the protective effect of social mobility belief is not general but rather depends on the context. Thus, the present study suggests that the function of SIDM may depend on context: under chronic stress, SIDM functions as a protective rather than a risk factor.

Our findings echo the broader literature on the paradoxical role of protective or risk factors in the ACEs context. For example, recent research revealed that some conventional protective factors (e.g., individual resilience attributes and perceived social support) even backfired, exacerbating the adverse psychosocial impacts of ACEs (48, 49). Taken together with our study, this further illustrates that neither protective nor risk factors are unidirectionally beneficial or harmful, their effects may exhibit pronounced context dependency, calling for a more flexible, context-sensitive understanding of the roles of protective and risk factors.

Indeed, contrary to our findings, SIDM was reported to amplify the negative links between ACEs and resilience (31), further supporting the context-dependent protection functionality of SIDM. ACEs represents a multidimensional stressor, including various forms of abuse and neglect (31), while EN is a component of ACEs and represents a relatively unidimensional stressor. Therefore, SIDM may exert distinct regulatory effects depending on stressor complexity. Integrating previous and present findings, the protective effects of SIDM appear to be EN-specific: SIDM may attenuate the negative connections between EN and PMH, whereas it exacerbates the negative relations between ACEs and PMH. Our findings underscore the potentially unique protective role of SIDM in the context of EN. Future research should examine the relationship between acute stressors and PMH, and differentiate types of childhood trauma to further elucidate this context-specific protective role of SIDM.

#### 4.3.2 The moderating role of stress-is-debilitating mindset between emotional neglect and stress-is-enhancing mindset

Hypothesis 4 was not supported by the finding that SIDM positively moderated the negative connections between EN and PMH via SIEM. Specifically, when SIDM was low, EN had a significant indirect negative link to PMH via SIEM. When SIDM was high, the indirect negative association between EN and PMH via SIEM was neutralized. That is, as the levels of SIDM increase, the negative relation between EN and PMH via SIEM decreases.

From a statistical perspective, under high SIDM, the direct effect of EN on PMH was significant, while the indirect effect of EN on PMH via SIEM was not significant. Hence, the current finding is not in accordance with the statistical suppression effect (50), and is more likely to reflect the individuals’ differences in chronic stress adaptation. De Raeymaecker et al. (51) claimed that the combination of multiple traits, rather than any single trait, determined whether an individual can benefit from instant messaging. Accordingly, our finding suggests that the combination of EN, SIDM and SIEM collectively determines whether an individual has good PMH. Specifically, the protective role of SIEM on PMH may only be effective for individuals with EN who have a low SIDM, while when individuals with EN who hold a high SIDM, SIEM’s protective role is neutralized.

In addition, it is reported that the indirect negative effect of childhood trauma on PMH via PMHL was weakest when both SIDM and SIEM were high (33). The current finding revealed a similar but slightly different pattern: when individuals hold high SIDM, the SIEM associated with EN is relatively high. However, this relative advantage of SIEM does not translate into the significant improvements of PMH. According to the cognitive dissonance theory (32), when individuals have low SIDM, their core belief of “stress has few negative effects” may create a cognitive conflict with the stronger emotional distress linked to EN. To achieve cognitive consistency, they may adjust their original belief of stress and adopt the new understanding that “stress is harmful”, thereby amplifying the negative association between EN and SIEM and negatively predicting subsequent PMH. In contrast, for individuals with high SIDM, the emotional distress associated with EN aligns with their core belief that “stress is harmful”. Hence, cognitive dissonance is avoided, the decline of SIEM linked to EN is attenuated. However, subsequent PMH is merely maintained at the baseline, and this maintenance does not yield a statistically significant indirect effect. This is in accordance with the motivation of SIDM to prevent adverse outcomes (23, 24).

Notably, our study reveals that, in the chronic trauma contexts, SIDM’s protective role may not function as maladaptive catastrophizing, but rather as adaptive threat appraisals. Specifically, children learn to understand and express emotions by observing parental stress responses, parental reactions to their emotional needs, and whether parents teach them to identify and express emotions (52). When chronically exposed to EN, children may develop a cognitive-emotional pattern of “emotions need no response”, and internalize it as a core negative metacognitive belief about the risk linked to emotional expression (i.e., SIDM). For example, “I should not express my emotions, because they will not be responded to”. From this lens, high SIDM may represent a shaped functional cognitive adaptation in chronic stress context——mitigating the emotional distress related to EN by reducing expectations of emotional response or suppressing emotional expression. The functional cognitive adaptation may be the mechanism by which individuals with high SIDM experience no cognitive conflict when facing the emotional distress linked to EN, weakening the adverse effects of EN on SIEM.

Our findings further suggest that, in the chronic stress adaptation process, SIDM and SIEM may not represent a simple antagonistic relationship but rather interact to influence the adaptive outcomes of individuals with EN. Recent network analysis indicates that parents’ positive and negative emotional responses during childhood interactively influence children’s emotional regulation, and the coexistence of positive and negative emotional behaviors is related to fewer emotional regulation difficulties (53). Existing evidence showed that SIDM is linked to maladaptive coping strategies (30), whereas SIEM is related to adaptive coping strategies (25, 29). Therefore, in the present study, SIDM and SIEM may respectively be associated with maladaptive and adaptive coping strategies, and these two types of strategies work together to alleviate emotion regulation difficulties in individuals with EN, thereby decreasing the negative effects of EN on PMH. In other words, SIDM may buffer emotional distress by suppressing emotional expression, while SIEM may maintain PMH from further deterioration through positive coping styles, the synergistic protective mechanism of SIDM and SIEM may be an intrinsic basis of chronic stress adaptation.

In summary, our finding shows that in the context of EN, SIDM may play an attenuating role in the negative effects of EN. However, the nature of this attenuating role is likely to reflect that the dominant adverse effect of SIDM overshadows rather than buffers the negative effect of EN on SIEM, therefore, it is insufficient to foster the positive psychological transformation needed for PMH.

#### 4.3.3 Implications and limitations

This study revealed that SIEM mediated the relation between EN and PMH, and this mediating path was moderated by SIDM. These findings provide important implications for psychological interventions targeting individuals with EN: interventions should not focus solely on directly enhancing SIEM, but more critically, on identifying and adjusting individuals’ levels of SIDM. For individuals with high SIDM, it is first necessary to create a sufficiently safe psychological space, affirm the protective function of SIDM, and then gradually reduce the levels of SIDM, allowing the positive resources opportunities to grow naturally. For individuals with low SIDM, threat awareness needs to be moderately enhanced to prevent blind optimism from being easily shattered by trauma. By coordinating both SIEM and SIDM to a more balanced state, SIEM can function effectively, truly achieving the goal of promoting psychological growth.

This study has several limitations. First, the current sample is recruited by convenience sampling from only four universities in East and Northeast China and it may lack sufficient representativeness. Especially, 83.53% of the present sample are sophomores, and fourth-year and higher students are entirely absent. Consequently, the present findings can be limited to lower-division undergraduates. From a perspective of development stages, the second year is a critical stage for identity development and interpersonal maturity, during which college students often encounter unique challenges (54). In contrast, upper-year students may typically have more developed identity and autonomy, and face different stressors (e.g., career preparation). However, the overrepresentation of sophomores in current sample may introduce a selection bias, making it difficult to reveal differences between different grade levels. Recent methodological articles emphasize the importance of stratified sampling across developmental stages, such as a diverse sample across grade levels (55). Therefore, future research should employ stratified sampling across grade levels to test whether the present findings broadly replicate in college students.

Second, the cross-sectional design can only reveal correlations among variables rather than establish causal relationships. Although we have examined whether EN has a negative effect on PMH by attenuating SIEM, the alternative direction cannot be ruled out. For instance, individuals with high PMH also may hold high SIEM, which in turn could affect how they perceive and retrospectively report EN. Therefore, future research should adopt longitudinal designs to test bidirectional or reverse causal models to clarify the temporal ordering among these variables. Notably, a three-wave study indicates that EN at T1 shows a negative link to gratitude at T3 (56), this finding directly specifies the connective direction between EN and PMH and highlights the need for temporal separation when examining the causal relationship between EN and PMH in the future.

Recently, a prospective design study measures the stress mindset, stress experience, and sleep outcomes at baseline and 1 year after. Results reveal that SIEM is positively associated with sleep outcomes 1 year later, independent of baseline sleep outcomes and stress levels (57), demonstrating that the causal relationship between SIEM and sleep outcomes. Although the prospective design did not directly measure EN and PMH, providing a causal methodological template and conceptual support for investigating whether SIEM as a mediator or moderator to buffer the negative effect of EN on PMH in the future. Furthermore, future studies could also employ longitudinal analysis strategies capable of distinguishing within-individual and between-individual differences—such as the random intercept cross-lagged panel model (58)—to further examine the causal relationships and dynamic patterns among SIEM, SIDM, EN, and PMH.

Third, the data in the current study were collected at a single time point. While the CMB was not severe in this study, it remains challenging to completely rule out the impact of CMB on the present findings. Future research should further employ temporal separation method to remove potential CMB effects. In addition, while the large sample size in this study, the observed interaction and moderated mediation effects are relatively modest. This finding suggests that SIDM may function as a moderator in the relationship between EN and PMH, but its practical effect is limited. Therefore, the findings of this study should be cautiously interpreted as preliminary evidence for SIDM as a moderator rather than the effects with strong practical significance. Although the limited practical effects of this study, small effects may yield meaningful aggregate impacts when operating across large populations (59). Therefore, future research should employ longitudinal designs and further validate these findings in diverse populations.

Finally, this study failed to uncover the specific psychological mechanisms through which SIEM exerts its mediating effect (e.g., challenge appraisals or adaptive coping strategies), future research could construct chain mediation models for in-depth exploration. Moreover, this study only examined the pathways of EN, while emotional abuse, as another form of emotional deprivation with more overt harmful manifestations, whether follows identical pathways awaits future investigation.

## 5 Conclusion

The current study preliminary identifies a conditional mediating mechanism via which EN affects PMH. EN is directly and negatively associated with PMH, but also indirectly and negatively associated with PMH through SIEM, high levels of SIDM fundamentally alter these links. Specifically, a high level of SIDM buffers the direct negative connection between EN and PMH and the indirect negative links between EN and PMH via SIEM. These findings highlight the importance of the interplay between SIEM and SIDM in chronic stress adaptation, suggesting that post-traumatic cognitive processing may follow a non-linear trajectory in which heightened risk awareness can paradoxically reframe early adversity as a potential catalyst for growth. Therefore, for individuals with EN, the interventions should not only cultivate SIEM but also leverage their SIDM as a starting point to guide cognitive restructuring, thereby facilitating growth.

## Data Availability

The raw data supporting the conclusions of this article will be made available by the authors, without undue reservation.
